# Characterizing spatial tuning functions of neurons in the auditory cortex of young and aged monkeys: a new perspective on old data

**DOI:** 10.3389/fnagi.2012.00036

**Published:** 2013-01-04

**Authors:** James R. Engle, Gregg H. Recanzone

**Affiliations:** ^1^Department of Psychology and Center for Neuroscience, University of California at DavisDavis, CA, USA; ^2^Evelyn F. McKnight Brain Institute and ARL Division of Neural Systems, Memory and Aging, University of ArizonaTucson, AZ, USA; ^3^Center for Neuroscience and Department of Neurobiology, Physiology and Behavior, University of California at DavisDavis, CA, USA

**Keywords:** auditory cortex, spatial tuning, lateral belt, macaque

## Abstract

Age-related hearing deficits are a leading cause of disability among the aged. While some forms of hearing deficits are peripheral in origin, others are centrally mediated. One such deficit is the ability to localize sounds, a critical component for segregating different acoustic objects and events, which is dependent on the auditory cortex. Recent evidence indicates that in aged animals the normal sharpening of spatial tuning between neurons in primary auditory cortex to the caudal lateral field does not occur as it does in younger animals. As a decrease in inhibition with aging is common in the ascending auditory system, it is possible that this lack of spatial tuning sharpening is due to a decrease in inhibition at different periods within the response. It is also possible that spatial tuning was decreased as a consequence of reduced inhibition at non-best locations. In this report we found that aged animals had greater activity throughout the response period, but primarily during the onset of the response. This was most prominent at non-best directions, which is consistent with the hypothesis that inhibition is a primary mechanism for sharpening spatial tuning curves. We also noted that in aged animals the latency of the response was much shorter than in younger animals, which is consistent with a decrease in pre-onset inhibition. These results can be interpreted in the context of a failure of the timing and efficiency of feed-forward thalamo-cortical and cortico-cortical circuits in aged animals. Such a mechanism, if generalized across cortical areas, could play a major role in age-related cognitive decline.

## Introduction

Auditory information is processed hierarchically in serial and parallel feed-forward pathways from the auditory thalamus to the core and belt areas of the auditory cortex (Hashikawa et al., 1991; Rauschecker, 1998; Hackett et al., 2001; De la Mothe et al., 2006). The auditory thalamus sends parallel afferent projections to auditory cortex that terminate in the thalamo-cortical recipient layer IV, cortico-cortical recipient layer II/III [for review, see Jones (2003)], and the cortico-thalamic recipient layer VI (Llano and Sherman, 2009). In primates, the direct primary pathway arises from the ventral division of the medial geniculate nucleus and terminates in layer IV of the auditory cortical core, while the tegmental pathway arises from the dorsal and medial divisions of the medial geniculate nucleus and terminates in layer II/III of core and the belt (Hashikawa et al., 1991; Molinari et al., 1995; Rauschecker et al., 1997). From these anatomical projections, Jones (2003) hypothesized that the parallel pathways to the core and belt fields form a coincidence detector circuit between the thalamo-cortical and cortico-cortical inputs, which suggests that the auditory processing specialization of areas in the belt field is dependent upon the coordinated activation of excitatory and inhibitory networks in and between the core and belt. Applying this hypothesis to spatial processing predicts that the serial refinement of spatial tuning curves observed between A1 and CL (Woods et al., 2006) is dependent upon the coordinated activation of excitatory and inhibitory networks that enhance the firing rate at the best location and/or suppress the firing rate at locations forming the flanks (i.e., away from the best location) of the spatial tuning curves. This predicts that there will normally be less activity due to more inhibition at the flanks away from the best location of the tuning curve in CL neurons compared to A1 neurons.

Recently, we have shown that compared to younger animals, A1 and CL neurons in aged macaques have higher spontaneous and driven activity and that the sharpening of spatial tuning between A1 and CL is degraded (Juarez-Salinas et al., 2010). One explanation for this is that there is an uncoordinated and unbalanced activation of excitation and inhibition into A1 and CL in the thalamo-cortical parallel pathway and/or the serial connections between A1 and CL. This finding would be consistent with both physiological and anatomical studies, largely in the rodent, that indicate a decrease in inhibition along the ascending auditory pathway as a consequence of aging [for review, see Caspary et al. (2008)] and that this decreased inhibition underlies central processing deficits in age-related hearing loss, including deficits in auditory spatial perception (Brown, 1984; Kubo et al., 1998; Abel et al., 2000). The present study re-examines these data in young (Woods et al., 2006) and aged (Juarez-Salinas et al., 2010) monkeys and investigates whether the differences previously found between the activity of neurons in A1 and CL in aged animals are due to differences in the firing rates that selectively affect different time periods within the response, for example by changes in on- or off-responses, and also if the firing rates are selectively affected for different locations within the spatial tuning curves.

## Materials and methods

### Animals

Five adult male rhesus macaque monkeys (*Macacca mullata*) were included in the study (for details, see Table 1). The young monkeys were 5.1–6.2 years old (monkey F), 6.3–7.5 years (monkey G), and 9.5–11.5 years old (monkey L), whereas the aged monkeys were 24.1–25.8 years old (monkey A) and 24.4–26.2 years old (monkey B) through the course of this study. Macaques age at approximately three times the rate in humans, so the aged animals are roughly the human equivalent of 75 years old. Neuronal data from all monkeys have been previously reported (Woods et al., 2006; Juarez-Salinas et al., 2010), except for an additional 23 neurons that were recorded in area A1 of monkey B. All monkeys used in this study had no history of exposure to chronic noise or known ototoxic substances, were free of known auditory impairments and had normal detection audiograms from 500 to 16000 Hz as well as to the broadband noise stimuli used here [see Juarez-Salinas et al. (2010)]. All experimental procedures conformed to the National Institutes of Health guidelines for animal use, and were approved by the UC Davis Institutional Animal Care and Use Committee.

**Table 1:** **Number of neurons recorded**.

**Monkey**	**Young**				**Aged**		
	**F**	**G**	**L**	**Total**	**A**	**B**	**Total**
A1	50	149	130	329	57	121	178
CL	71	0	119	190	42	49	91
Total	121	149	249	519	99	170	269

### Stimuli and apparatus

Each monkey was trained to sit quietly in an acoustically transparent primate chair (Crist Instruments) while listening to auditory stimuli presented at head level from 1 of 16 speakers spaced at 22.5° that spanned 360° in azimuth. A 200 ms duration “unfrozen” Gaussian noise was presented at 55 and 75 dB SPL (5 ms rise/fall) to the young monkeys, while a 200 ms duration “unfrozen” Gaussian noise was presented at 65 dB SPL to the aged monkeys. No differences were found between the 55 and 75 dB SPL conditions in any of the analysis reported here, so the responses from both conditions were pooled [see Woods et al. (2006)]. A minimum of 12 samples from each speaker were recorded per experimental session with an inter-trial-interval of about 5 s. Additionally, 12 trials with no sound were randomly interleaved with the acoustic stimuli, and were used to define spontaneous activity. A trial was initiated by depressing and holding a lever while several stimuli (3–7) were presented randomly from different speakers (S1) until a stimulus was presented from the same speaker twice (S2). A small fluid reward was then provided. Two of the younger monkeys (F and L) then released the lever to receive a larger fluid reward, while the younger monkey G and the two aged animals (A and B) sat quietly but heard the same sounds and received the same two rewards at about the same interval. Our previous analysis showed no difference between the three young monkeys, but a clear difference between monkey G and the two aged animals (Juarez-Salinas et al., 2010), which indicates that these task differences did not influence spatial tuning, spontaneous, or driven activity. Nonetheless we considered the possibility that the two younger animals were actively localizing these sounds, which subsequently could influence their response properties. To test this possibility we calculated the hit rate for these two monkeys as a function of the location and intensity of the stimulus that immediately preceded the reward delivery. For this analysis, we also included behavioral data for stimuli presented at 25 dB SPL and 35 dB SPL (Woods et al., 2006). Both monkeys showed very high performance rates (between 96.8–99.4% and 89.9–94.1% in monkeys L and F, respectively). ANOVA analysis revealed that there was no effect of location on this performance [*F*_(15, 640)_ = 0.195, *p* > 0.05], but there was a significant effect of intensity [*F*_(3, 640)_ = 4.700, *p* < 0.01] with the highest intensity stimulus resulting in slightly lower performance compared to the three lower intensity stimuli (97.1 vs. 99.1% for monkey L and 90.8 vs. 92.8% for monkey F for high and low intensity stimuli, respectively). This finding opposes what has previously been observed in humans and monkeys, where low intensity stimuli were not as well localized as high intensity stimuli (e.g., Su and Recanzone, 2001; Recanzone and Beckerman, 2004; Sabin et al., 2005), and are opposite to humans using these same acoustic stimuli, where performance was statistically significantly worse for the lowest intensity stimuli as well as for locations in the rear quadrant (Miller and Recanzone, 2009). Thus, these animals were likely not using spatial information when performing the task and this result is consistent with our previous findings that the subtle differences in the behavioral task did not significantly contribute to any of the effects studied.

## Recording procedures

A recording cylinder and head post were surgically implanted to allow for a vertical approach to the superior temporal plane of the superior temporal gyrus [for detailed methods, see Recanzone et al. (2000)]. During each recording session, a tungsten microelectrode (FHC, 2–4 Mohms) was inserted into a guide tube that penetrated the brain by ~3 mm to prevent microelectrode bending, and was advanced by a hydraulic microdrive (Narishigi, JP) into auditory cortex. Neural activity was displayed on an oscilloscope and audio monitored. Search stimuli consisted of broadband noise bursts, tones, band-passed noise, and clicks. Single neurons that could be driven were isolated using a time-amplitude window discriminator (BAK). The time of occurrence of each action potential was time stamped at a resolution of 1 ms. Neurons were assigned to either A1 or CL based on the location within the recording cylinder, frequency tuning and other response properties, and the progression of characteristic frequency across the recording cylinder. These locations were verified histologically in all animals except monkey B, which is still participating in experiments [see Juarez-Salinas et al. (2010) for further details].

## Data analysis

Individual neuronal post-stimulus time histograms (PSTHs) were constructed by summing the spike count over each of the trial repetitions using 1 ms time bins for each speaker location within each recording session. We defined the best and worst direction as the speaker location within our speaker array that evoked the greatest and the lowest responses that were statistically different from spontaneous activity (paired *t*-test, *p* < 0.05, Bonferroni corrected), which was acquired during the no-stimulus control condition. Early (0–100 ms), late (100–200 ms), and off (225–275 ms) response periods were analyzed to examine the different periods of the neuronal response.

Since acoustic space is not topographically organized in the primate auditory cortex (Recanzone et al., 2000; Miller and Recanzone, 2009), we normalized acoustic space to the best direction in each neuron and then summed them to analyze the population response. Population PSTHs were constructed by pooling the activity of each corresponding 1 ms bin across recording sessions, and then were smoothed by averaging across a 5 ms window throughout the entire recording period. It is plausible that neuronal responses are inhibited throughout the stimulus presentation window from all speaker locations. This activity pattern, however, was not encountered in our dataset as we collected data from neurons that were initially identified as driven by our search stimuli.

Spike rate was then calculated across the three different neuronal response periods without compensation for the travel time from the speaker to the tympanic membrane, and followed the parameters provided by Recanzone (2000). The driven firing rate for each period was defined as the averaged neuronal response minus the averaged spontaneous activity for each period. Spontaneous activity was defined as the averaged neuronal activity during the control no-sound condition, and was calculated across the three response periods mentioned above. Excitatory responses were defined as driven firing rates greater than 0, while inhibitory responses were defined as driven firing rates less than 0. We measured inhibitory responses during the early, late, and off response periods by calculating the percent of neurons that had driven firing rates less than 0.

During each response period the rate, temporal, and inhibitory spatial tuning indices were calculated to measure the depth of spatial tuning curve properties. For the metrics listed below, values of 0 indicate that the response was not spatially tuned, and values become more positive as the spatial tuning increases. We used a firing rate-based spatial tuning index (RTI) that quantifies the shape of spatial tuning curves that is encoded by the driven firing rates, and is calculated by:

$$\text{RTI}=1-({\left(\text{Response }\Delta \right)}^{2}/({\text{Response}}_{\text{best direction}}{-{\text{Response}}_{\text{worst direction}})}^{2})$$

where Response Δ is calculated by:

$$\text{Response }\Delta =\sum ({\text{Response}}_{\text{best direction}}-{\text{Response}}_{i\text{th direction}})\text{/n direction}s$$

A value of 1 indicates a flat spatial tuning. Larger values that approach 1 indicate broad spatial tuning, whereas smaller values that approach 0 indicate narrower spatial tuning curves.

First-spike latencies were measured from the population PSTHs. Population excitatory first-spike latency was defined as the first bin with at least 5 consecutive bins that were significantly greater (paired *t*-test, *p* < 0.01) than baseline (the first 5 ms of the recording period). This technique can also be applied to individual neuronal responses within each cortical area. To identify pre-onset inhibition, inhibitory latency was defined more liberally as the first bin and any consecutive bin that was significantly lower (paired *t*-test, *p* < 0.01) than baseline. This strategy is a robust measure that avoids biases generated by transient spontaneous activity, response heterogeneity across directions, and instances when the baseline condition is equal to 0. We used an analogous metric of the RTI, the latency-based tuning index (LTI), to assess the dynamic range of spatial tuning that is encoded by the latency of the response, and is calculated by:

$$\text{LTI = 1}-\;\left({\text{Latency}}_{\text{best direction}}{\text{/Latency}}_{\text{worst direction}}\right)$$

We then used a rate-based inhibition tuning index (ITI) to determine how the dynamic range of spatial tuning is encoded by neuronal inhibition, and is calculated by:

$$\text{ITI }=\;\left({\text{Response}}_{\text{worst direction}}-\text{Spontaneous Response}\right)\text{​}/\left({\text{Response}}_{\text{worst direction}}\text{+ Spontaneous Response}\right)$$

In this case, excitatory responses result in positive values approaching 1, responses the same as the spontaneous rate result in values of 0, and inhibited responses result in negative values approaching –1. Statistical analyses were performed with SPSS, version 20 (Chicago, IL). MANOVA and mixed ANOVA were used to analyze for differences in driven firing rate, RTI, and ITI from neural responses in both A1 and CL of young and aged monkeys. The Tukey multiple comparison test was used to identify significant differences when one or more differences were found. Chi-squared and linear regressions were used to test for changes in first-spike latency measurements. Multiple comparisons were corrected by the Bonferroni method, and all significant differences were considered significant at an adjusted *p*-value less than 0.05.

## Results

**Figure 1:**
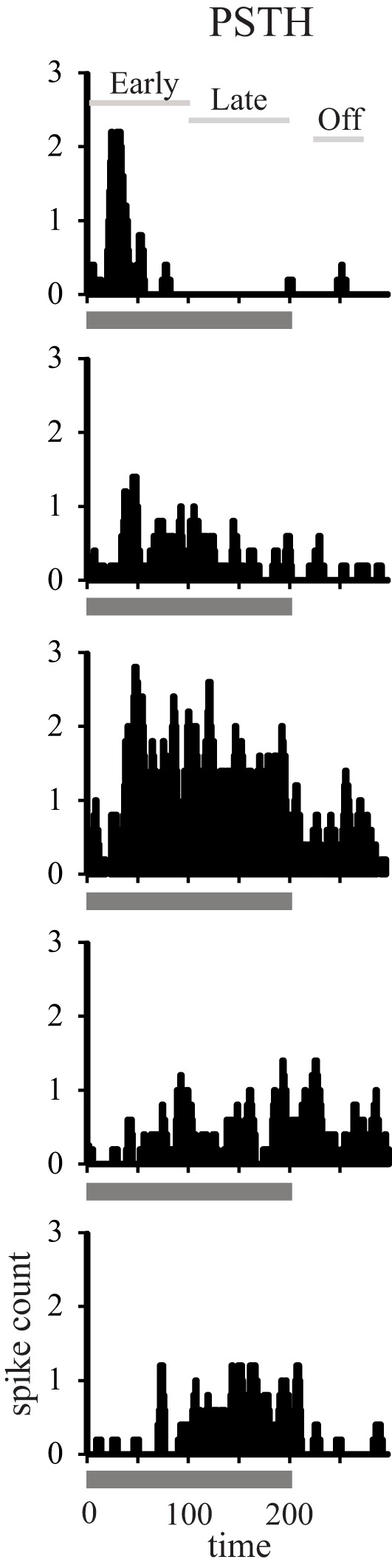
**Temporal response profiles of neuronal responses.** Recorded neuronal responses varied along a continuum from early phasic (top), tonic (middle), to the late period only (bottom).

Using standard single-unit recording techniques, we recorded the neuronal activity elicited by broadband noise bursts of 788 single neurons in auditory cortical areas A1 and CL in both young and aged alert monkeys (Table 1). Figure 1 shows five representative examples of the types of responses we encountered in both young and aged animals. We encountered neurons with a short duration onset response, those with a smaller or even absent onset response but a clear tonic response throughout the stimulus presentation, and those that only fired during the last part of the stimulus. This continuum is represented down each column of Figure 1, and as described previously in young animals in A1, forms a relatively uniform distribution (Recanzone, 2000).

**Figure 2:**
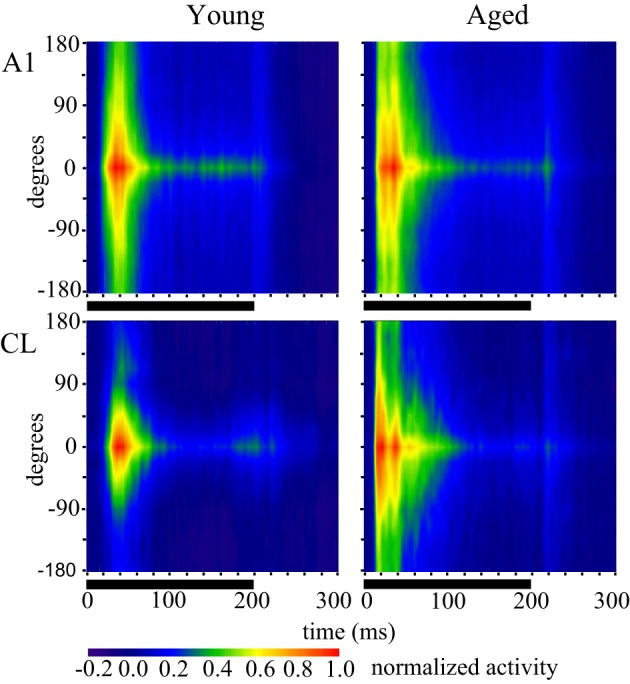
**Population response across time and spatial location.** Each panel shows the response of the population of A1 (top) and CL (bottom) neurons in young animals (left) and aged animals (right). Warmer colors represent greater activity. The response period is shown as the solid line below each panel. Each neuron was normalized to the best direction at 0 along the y-axis, with leftward eccentricities below and rightward eccentricities above. Young animals showed an increase in spatial tuning between A1 and CL, as shown by less activity beyond about ± 90°. There was also more of a sustained response for the best direction compared to eccentric directions. Aged animals showed broader tuning in general, particularly in area CL, and a more oscillating response over time.

In order to generalize these responses as the population response within a given cortical area and age group we generated population heat maps of the response as a function of stimulus location and time. For each individual neuron, we normalized the location with the highest firing rate to location 0. We then summed the activity of each 1 ms bin for each neuronal recording session across all neurons recorded in that cortical area in young or aged animals, so that each bin across all acoustic directions and time are represented by a minimum of 1000 trials that accrued across recording sessions. Next, we normalized the population response to the peak response in the best direction, and then used colors to represent the normalized activity. The cortical representation of broadband noise across 360° of acoustic space from the population of neurons A1 and CL in young and aged monkeys is summarized in Figure 2, with warmer colors representing higher firing rates. One interesting feature that is clearly apparent when the data are represented in this way is the temporal dynamics of the response between young (left column) and aged animals (right column). Young animals have an initial burst of activity that decreases consistently with time for stimuli at any given location, and a decrease in activity as the location moves away from the best direction. In contrast, in aged animals there are clearly two peaks of activity across locations and at least three at the best direction. This activity also persists longer in the aged animals compared to younger animals. A second obvious qualitative feature is that the responses in young animals are more sustained for the best direction compared to the flanking directions, particularly for CL neurons. In aged animals the sustained activity is not as dependent on the spatial location of the stimulus as in younger animals. A third notable feature is that the latency for this activity is shorter in aged animals compared to younger animals. Finally, there are clear differences in the responses between the first 100 ms and the second 100 ms of the stimulus period, as well as an increase in activity shortly after the stimulus ended.

**Figure 3:**
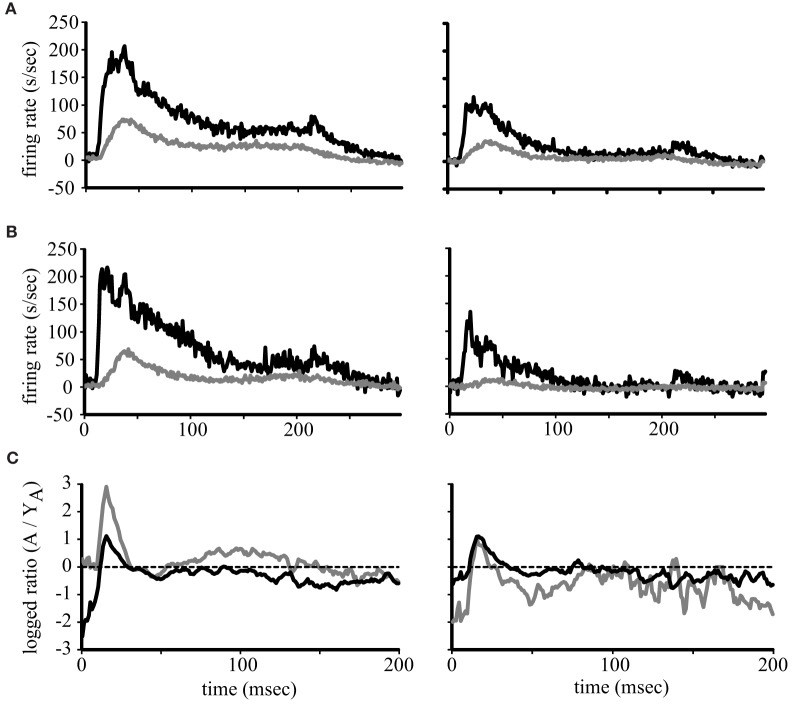
**Population firing rates.** Each line represents the average firing rate across the population of neurons in young (gray lines) and aged (black lines) monkeys for A1 **(A)** and CL **(B)**. The response to the best direction is to the left and the worst direction to the right. Panel **(C)** shows the logged ratio between young and aged monkeys during the first 200 ms of the response.

The above representation of acoustic space used a normalization procedure, which loses the magnitude of the responses. Our previous study showed that driven activity is higher in aged monkeys compared to younger ones (Juarez-Salinas et al., 2010) but that analysis was restricted to 350 ms after stimulus onset so whether any particular portion of the response was selectively enhanced or suppressed was not addressed. Given the differences in the firing rates across the population between the early and late period of the stimulus (Figure 2) we therefore examined the population temporal response profiles of young and aged monkeys for both A1 and CL neurons. The population temporal response profile was constructed by collapsing each individual PSTH into a population response by summing the activity of each 1 ms bin across neuronal recording sessions and dividing by the total number of neurons. The results are shown in Figure 3, where the population response in the best direction is shown in the left column, and the response for the worst direction is shown in the right column. Panel (3A) shows the results from A1 neurons, with neurons from young animals shown as the gray lines and neurons from aged animals as the black lines. There is a clear increase in activity in the aged animals, but this increase is most dramatic near the onset of the responses in both the best and worst directions. A similar finding was noted for the population of CL neurons (Panel 3B) where there appeared to be two peaks of activity in the best direction; an initial peak and a second that could potentially correspond to the initial peak in the younger animals. To quantify these impressions we examined the log_2_ of the ratio between the population response in young monkeys to the population response in aged monkeys in both area A1 and CL and in the best and worst directions after normalization. We normalized the population response of the young monkeys to the population response in aged monkeys by multiplying by the peak difference between young and aged monkeys. This normalization occurred at the exact time that corresponded to the peak response found in the young monkeys. This strategy allowed us to overcome the large difference in overall activity to therefore illustrate differences in the shapes of the response between the two groups of animals, and express the magnitude of this difference between young and aged monkeys. Additionally, this analysis also gives us the ability to detect segments of the response that would normally be masked by inhibition (Calford et al., 1993; Norena and Eggermont, 2003; Norena et al., 2003). Values of 0 indicate no difference between the normalized young population responses to the aged population response, and positive values indicate that segments of the response that was greater in the aged monkeys compared to the young ones. These results are shown in Figure 3C for A1 (black lines) and CL (gray lines) in the best (left) and the worst (right) directions. This normalization procedure elevated the peak response of young monkeys to comparable rates found in aged monkeys for the best direction. This can be observed between 40 and 50 ms in both A1 and CL, where both curves are at a value of 0. The normalization procedure, however, normalized the slight variation of the pre-onset spontaneous activity in CL, but not in A1. This is observed very early in the response between 0 and 10 ms. In A1, the segment of the response that was differentially excited was the initial onset response in both the best and worst direction. The response in CL had two segments that were differentially excited, which was an early segment of the onset response and a middle segment of the response.

## Age-related increase of driven firing rates during the early, late, and off periods

**Figure 4:**
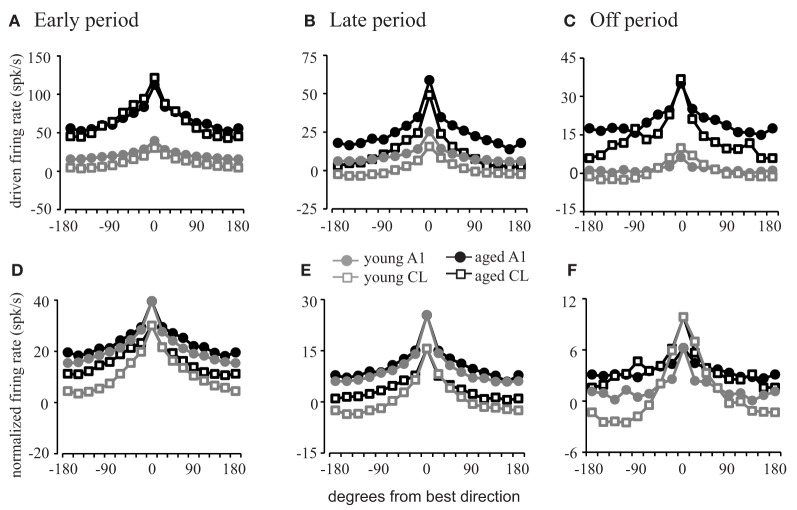
**Firing rates as a function of stimulus location.** Population firing rate curves relative to the best direction of each cell. **(A–C)** Show the absolute firing rate; **(D–F)** show rates normalized to the best direction of the young animals. Response profiles in the early **(A,D)**, late **(B,E)**, and off **(C,F)** periods are shown. Aged animals have a much greater driven firing rate than young animals **(A**–**C)**. The difference in the shape of the tuning functions is minimal for A1 neurons (circles) except in the off period. However, the tuning functions are much sharper in young CL compared to aged CL (squares) across all three response periods. See Table 2 for SEMs.

The previous analyses revealed that there is a higher firing rate in neurons from aged animals compared to those from young animals and that the response becomes less sustained as the stimulus moves away from the best direction. To provide an additional layer of quantification, we examined the driven response during the early, late, and off response periods as a function of degrees from the best direction in A1 and CL of young and aged monkeys. In order to examine how the overall firing rate influenced spatial tuning during the three different response periods, the average neuronal response was calculated by averaging the driven firing rate minus the spontaneous rate for all neurons recorded at each direction. Figure 4 illustrates the average firing rate spatial tuning curves during the early, late, and off response periods.

In order to explore these differences across spatial locations and to determine the degree of flanking inhibitory activity influence on the dynamic range of spatial tuning curves, we compared the averaged firing rates during each of the response periods with each neuron's tuning function centered on the best direction (Figure 4; see Table 2 for standard errors of the mean).

**Table 2:** **Standard errors of the mean for firing rates as a function of stimulus location (see Figure 4 for the mean values)**.

**Loc:**	**−180**	**−157.5**	**−135**	**−112.5**	**−90**	**−67.5**	**−45**	**−22.5**	**0**	**22.5**	**45**	**67.5**	**90**	**112.5**	**135**	**157.5**
**A1 EARLY**																
Young	0.93	0.94	0.99	1.02	1.09	1.14	1.22	1.27	1.48	1.27	1.21	1.14	1.03	1.01	0.95	0.94
Old	5.40	4.02	3.97	4.20	3.99	4.50	4.94	4.95	5.80	4.90	4.81	4.74	4.19	4.58	4.32	3.87
**A1 LATE**																
Young	0.72	0.74	0.71	0.74	0.79	0.82	0.89	0.91	1.17	0.90	0.85	0.80	0.73	0.77	0.71	0.70
Old	3.37	2.43	2.42	2.81	2.46	2.80	3.12	3.37	4.44	3.24	3.15	2.92	2.68	2.49	2.56	2.22
**A1 OFF**																
Young	0.87	0.82	1.02	0.91	0.78	1.22	0.84	0.87	0.99	0.86	0.83	0.92	0.82	0.92	0.79	0.86
Old	3.66	2.54	2.66	2.79	2.68	2.85	2.98	3.08	3.56	3.04	2.88	2.79	2.65	2.55	2.76	6.67
**CL EARLY**																
Young	0.58	0.54	0.54	0.59	0.70	0.88	1.04	1.11	1.26	1.16	1.00	0.92	0.82	0.69	0.66	0.62
Old	4.16	3.92	4.56	5.43	5.25	5.57	6.23	6.79	7.55	6.38	6.30	3.38	5.03	5.22	4.14	4.24
**CL LATE**																
Young	0.47	0.41	0.43	0.40	0.43	0.55	0.68	0.76	0.98	0.83	0.64	0.52	0.53	0.48	0.47	0.49
Old	2.62	2.48	2.44	3.02	3.82	3.15	3.72	4.13	5.36	4.29	3.53	2.85	3.47	2.84	2.50	2.63
**CL OFF**																
Young	0.70	0.68	0.68	0.70	0.66	0.72	1.11	0.82	1.00	0.90	0.80	0.80	0.78	0.67	0.68	0.70
Old	3.79	4.20	4.46	4.05	4.41	4.01	4.21	5.24	5.67	4.29	4.27	2.95	4.39	4.33	4.22	4.34

This analysis shows that the firing rate is elevated in all directions across each response period in both A1 (circles) and CL (squares) for both young (gray) and aged (black) neurons in the early period (Figure 4A), late period (Figure 4B), and off period (Figure 4C). During each of these periods and across directions, the firing rates of the aged neurons were greater than those of the young neurons in A1 [*F*_(1, 1274)_ = 356.986, *p* < 0.001] and CL [*F*_(1, 1274)_ = 210.664, *p* < 0.001], in some cases by several-fold. What is not clear from this figure, however, is if there is a change in the shape of the tuning curves themselves as a function of age. If flanking inhibitory activity is important for sharpening the spatial tuning curves, then this should be revealed by normalizing the responses to account for the increased firing rate in the aged monkeys. This is shown in panels (4D–F), where the responses of the aged neurons were normalized to that of the young neurons. In this case, there is very little difference in the shapes of the spatial tuning functions between the young and aged monkeys in A1 during all three response periods as indicated by the similarity of the tuning curves illustrated as circles. The apparent differences were that the aged neurons had a tendency to be a bit shallower (firing rates of aged animals are higher at non-best directions). In contrast, the spatial tuning functions of young CL neurons (squares) were much sharper than those found in aged CL neurons in all three periods, particularly during the early and offset periods. These differences were small at locations near the best direction, but locations eccentric to this rapidly decreased in the young animals compared to the aged animals. What can also be noted in this figure is that the overall shapes of these tuning functions are equivalent in aged monkeys between A1 and CL, whereas in younger monkeys the spatial tuning curves are much sharper in CL compared to A1. These results indicate that the increased firing rate observed in aged animals are relatively constant across spatial locations, and that the reduction in the response at the non-best directions is less apparent, particularly in CL. This result is consistent with a decrease of inhibition in the non-best directions across all portions of the response period in the aged animals.

**Figure 5:**
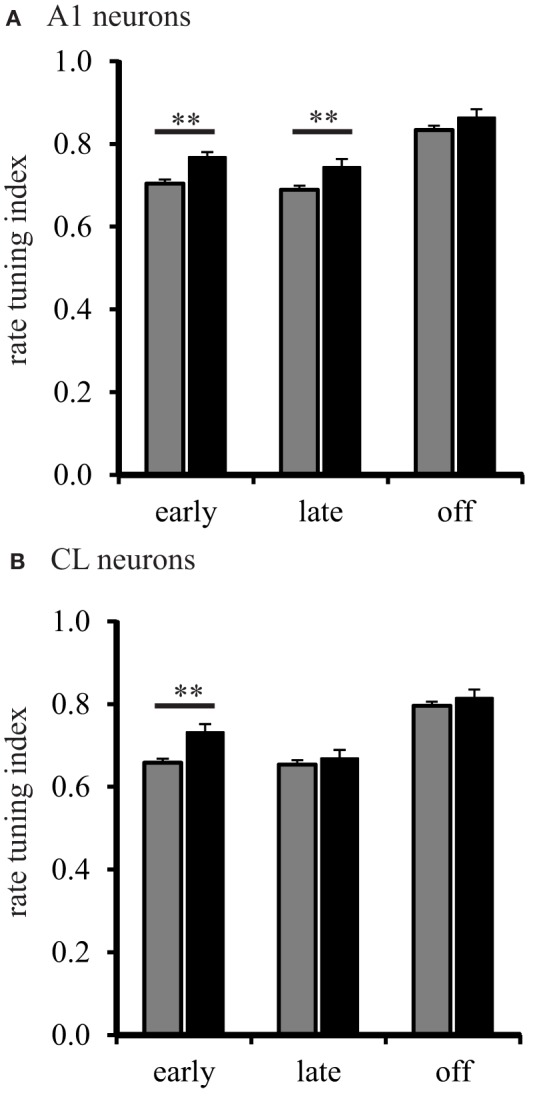
**Population spatial tuning index during different response periods.** Panel **(A)** shows the RTI during the early, late, and off periods in A1 of young (gray) and aged (black) monkeys. There were differences found except during the off period. Panel **(B)** shows the results from CL neurons. Here, aged monkeys were more broadly spatial tuned during the early period (^**^p < 0.01).

We then analyzed the shape of the spatial tuning curves from each neuron with the RTI. The results of this analysis are shown in Figure 5. The RTI of neurons in A1 of young monkeys gradually changed across the early, late, and off response periods, *F*_(2, 1275)_ = 131.01, *p* < 0.001. We found that this effect was driven by a significant increase (broadening) between the early and off, and the late and off response periods (Tukey Test, *p* < 0.001). The same effect was found for CL neurons, *F*_(2, 1275)_ = 76.867, *p* < 0.001. We found that spatial tuning in CL neurons also became significantly broader between the early and off, and late and off response periods (Tukey Test, *p* < 0.001). These results suggest that the spatial tuning of A1 and CL neurons are more sharply tuned following stimulus onset, and gradually become broader over the stimulus period. The data from A1 neurons in aged monkeys followed a similar pattern. Spatial tuning was found to gradually increase across the early, late, and off response periods, *F*_(2, 1275)_ = 22.52, *p* < 0.001. We found that the spatial tuning of A1 neurons became significantly broader between the early and off, and late and off response periods (Tukey Test, *p* < 0.001). A different pattern, however, was found in CL neurons of aged monkeys. Spatial tuning was found to be different across all response periods, *F*_(2, 1275)_ = 13.61, *p* < 0.001. This time, however, we found that the spatial tuning of CL neurons in aged monkeys was significantly lower (sharper) during the late period than during the early and off periods (Tukey Test, *p* < 0.001). These results suggests that A1 neurons in aged monkeys follow a similar pattern of gradually becoming broadly tuned over the stimulus period; whereas, CL neurons in aged monkeys follow a refinement pattern that is different from A1 neurons.

Next, we examined the shape of spatial tuning curves between A1 and CL to determine how sharpening of spatial tuning curves is modulated across the stimulus period between A1 and CL. In young monkeys, we found that the shape of spatial tuning curves of CL neurons were consistently sharper than A1 neurons during the early [*F*_(1, 1276)_ = 19.38, *p* < 0.001], late [*F*_(1, 1276)_ = 6.624, *p* < 0.01], and off [*F*_(1, 1276)_ = 7.03, *p* < 0.01] periods. In aged monkeys, we found that the shape of spatial tuning curves of CL neurons were only significantly sharper during the late period of the response, *F*_(1, 1276)_ = 7.71, *p* < 0.01. These results suggest that CL neurons may experience an age-related decrease in the enhancement or suppressive mechanisms that sharpen and refine spatial tuning functions between area A1 and CL. To confirm this impression, we then examined for age-related differences within A1 and CL. For A1 neurons, the shape of spatial tuning curves in aged monkeys was significantly broader during the early [*F*_(1, 1276)_ = 22.03, *p* < 0.001] and the late [*F*_(1, 1276)_ = 10.39, *p* < 0.001] periods. For CL neurons, the shape of spatial tuning curves in aged monkeys was only significantly broader during the early period, *F*_(1, 1276)_ = 14.16, *p* < 0.001. These results suggest that either inhibitory or excitatory mechanisms that facilitate sharpening of spatial tuning curves between A1 and CL are greatly impaired by age.

## Age-related decline of inhibitory activity as a function of spatial tuning

**Figure 6:**
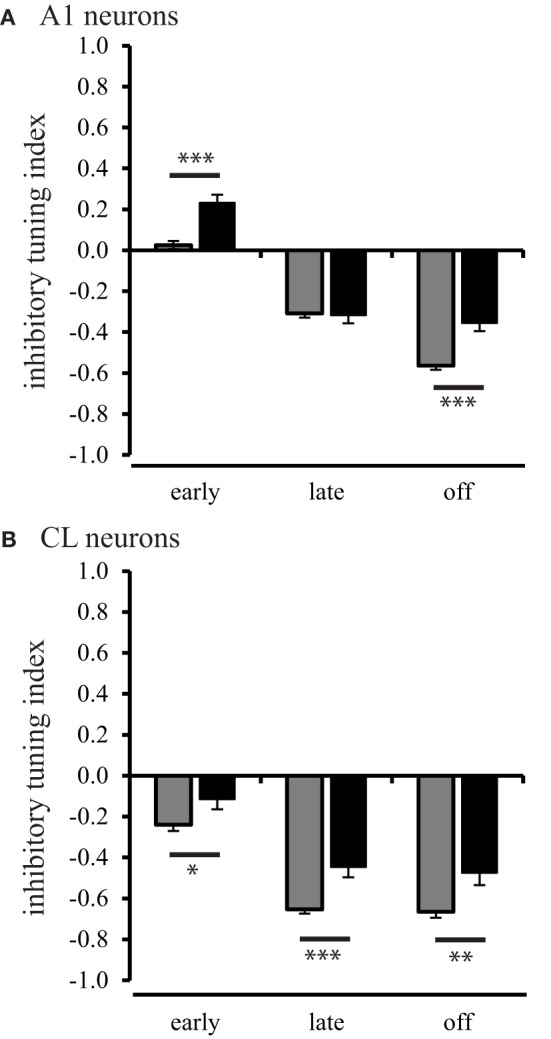
**Population inhibitory tuning index comparison between young and aged animals.** The inhibitory index showed a significant difference between young and aged A1 neurons during the early and off response periods (Panel **A**). Panel **(B)** shows that the strength of inhibition or suppressed response is greater in young animals compared to aged animals for CL neurons (^*^p < 0.05; ^**^p < 0.01; ^***^p < 0.001).

In order to investigate the inhibitory processes across spatial locations in neurons from aged and young animals, we first examined how much relative inhibitory activity was present across the population of neurons in the worst direction during the three different response periods. We used the rate-based ITI to quantify the strength of a suppressed response, defined as a response that was less than the spontaneous rate. ITI values range from −1 for strongly suppressed responses to 1 for strongly excitatory responses. ITI values greater than and equal to 0 indicate that the response in the worst direction was equal to the spontaneous rate (see Methods). The results of this analysis are shown in Figure 6. In A1 neurons of young monkeys, the strength of inhibition or suppressed responses continuously increased (Tukey Test, *p* < 0.001) over the early, late, and off response periods, *F*_(2, 1275)_ = 259.34, *p* < 0.001. In CL neurons of young monkeys, the increase in strength of inhibition or suppressed responses was restricted, however, to the early and late period (Tukey Test, *p* < 0.001) of the response, *F*_(2, 1275)_ = 119.94, *p* < 0.001. This finding is consistent with our data presented in Figures 2 and 3, which shows a response decrement over the response period. In the second layer of this analysis, we examined the strength of inhibition during the early, late, and off response periods between A1 and CL to determine if greater inhibition or response suppression contributes to changes along the cortical hierarchy. In young monkeys, we found that the strength of inhibition was consistently stronger on CL neurons than on A1 neurons across the early [*F*_(1, 1276)_ = 71.66, *p* < 0.001], late [*F*_(1, 1276)_ = 126.96, *p* < 0.001], and off [*F*_(1, 1276)_ = 9.10, *p* < 0.01] response periods. This indicates a greater inhibition or response suppression in the worst direction along the cortical hierarchy. In the next layer of the analysis, we examined if the strength of inhibition decreased during the early, late, and off response periods as a function of age for neurons in A1 (Figure 6A) and CL (Figure 6B). We found an age-related decrease for A1 neurons (Figure 6A), where aged neurons in A1 were less suppressed during the early [*F*_(1, 1276)_ = 25.95, *p* < 0.001] and off [*F*_(1, 1276)_ = 23.31, *p* < 0.001] periods. We also found an age-related decrease in the strength of inhibition on suppressed responses in CL neurons (Figure 6B), where aged neurons in CL were consistently less suppressed during the early [*F*_(1, 1276)_ = 4.62, *p* < 0.05], late [*F*_(1, 1276)_ = 13.22, *p* < 0.001] and off [*F*_(1, 1276)_ = 9.24, *p* < 0.01] periods. This suggests that there is an age-related imbalance of inhibitory and excitatory activity on the regulation of the neural response in the worst direction.

**Figure 7:**
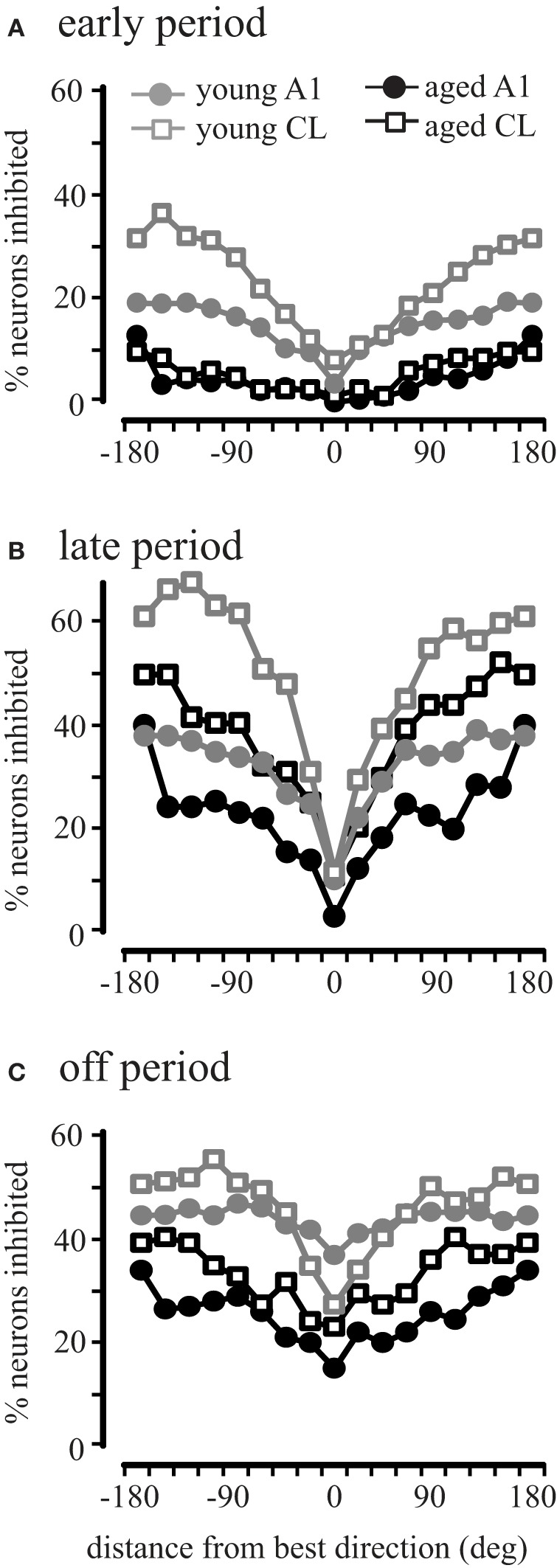
**Percentage of neurons inhibited during different periods of the response.** Very few cells showed inhibition during the early period of aged monkeys in both A1 and CL (Panel **A**) compared to younger animals. Similar results were seen for the late period **(B)** and the off period **(C)**.

This result across the population could be due to all neurons showing inhibitory activity in aged animals, or that there is a larger subset of inhibited neurons in aged animals. To investigate this we compared the percent of inhibited neurons in area A1 and CL of young and aged monkeys. Inhibitory responses were defined as driven firing rates during the early, late, and off that were less than the recorded spontaneous rate (Recanzone, 2000). Figure 7 illustrates the percentage of neurons inhibited in areas A1 and CL of young and aged monkeys as a function of degrees from the best direction during the early, late, and off response periods. In young monkeys, the percentage of neurons inhibited varied as a function of response period and degrees from the best direction. Regardless of the response period, the percent of inhibited responses increased as a function of degrees from the best direction. However, the percentage of inhibited responses in aged monkeys followed a slightly different pattern across the early, late, and off response periods in A1 and CL. We found a significant age-related decline for all directions in A1 (gray vs. black circles) during the early and off periods (χ^2^, *p* < 0.05), with only two locations showing a significant decline during the late period (157.5 and 180°). A similar result was seen in CL (gray vs. black squares), although there were more locations that did not show a significant age-related difference (early period, 0°, 22.5°, 45°; late period, −22.5°, 0°, 22.5°, 67.5°, 90°, 112.5°, 135°, 157.5°, 180°; off period, −22.5°, 0°, 22.5°, 45°, 67.5°). This finding indicates that there is much less inhibitory activity in aged A1 and CL neurons during the early period compared to younger animals, but also that inhibitory activity during the late and offset periods is more prevalent than during the early period in both groups of monkeys. These results indicate that younger animals show more neurons that are inhibited across directions and periods compared to aged animals.

## Age-related changes in the latency of the neuronal response

We have previously shown that spatial processing is degraded from area A1 to CL in aged monkeys based on the entire response period (Juarez-Salinas et al., 2010). However, auditory information is also processed in parallel streams from the auditory thalamus to auditory cortex (Hashikawa et al., 1991; Rauschecker et al., 1997; Rauschecker, 1998; Hackett et al., 2001), and these parallel pathways may form a coincidence detector circuit (Jones, 2003). A decline of inhibition may perturb the balance and coordinated arrival of excitatory and inhibitory inputs that shape and refine spatial tuning curves between A1 and CL, thus leading to degradation of spatial processing in CL. If the balance and coordinated arrival of excitatory and inhibitory inputs are perturbed by age, then excitatory and inhibitory first-spike latencies will be different in aged monkeys compared to young monkeys. To test this hypothesis, we calculated the first-spike latency by performing a paired *t*-test between the firing rate of the population of neurons during a 5 ms moving window compared to the first 5 ms of the response (see Methods). This eliminates the inherent difficulty in defining which spike is actually driven in neurons with high spontaneous activity, yet still accurately represents the population average. In young monkeys, the average population first-spike latency across locations was significantly faster (*t*-test, *p* < 0.05) in A1 (mean = 16.76 ms, SEM = 0.19) than in CL (mean = 19.76 ms, SEM = 1.03). In aged monkeys, no difference was found (*t*-test, *p* > 0.05) in the population first-spike latency between A1 (mean = 13.94, SEM = 0.15) and area CL (mean = 13.76, SEM = 0.11). However, we found a significant age-related decrease in the population first-spike latencies in A1 and CL (*t*-test, *p* < 0.01). These findings suggest that on average there is a slight delay between the excitatory first-spike in A1 and CL, and that the mechanism that generates this delay is degraded in aged monkeys.

**Figure 8:**
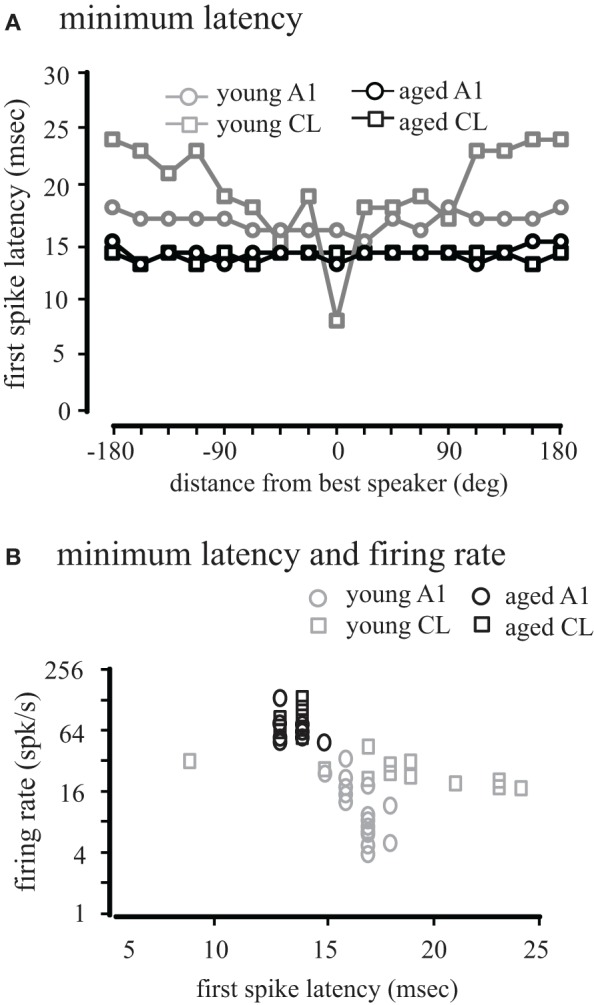
**Population first-spike latency.** The first-spike latency at each location relative to the best direction is shown for young (gray) and aged (black) monkeys in A1 (circles) and CL (squares) in Panel **(A)**. Aged monkeys had consistently shorter first-spike latencies than young animals. There was also no difference between A1 and CL in aged monkeys, but in young animals latency increased with distance from the best location. **(B)** Shows the regression between minimum latency and firing rate. There was a significant increase in latency with a decrease in firing rate in younger animals, but this correlation did not exist in aged animals.

We also considered how first-spike latency was related to the spatial location of the stimulus. Figure 8A shows the minimum first-spike latency across the population of neurons as a function of distance from the best direction. In young monkeys, there was a slight increase in latency in A1 neurons with increasing distance from the best direction (gray circles). In contrast, for CL neurons there was a much greater increase in latency when stimuli far from the best direction were presented (gray squares). In contrast for both A1 and CL in aged animals (black symbols) the latency was largely invariant across spatial location in both A1 and CL, indicating that early inhibitory activity is normally spatially dependent in CL, and this is lost in aged animals.

This latency data is consistent with the spatial tuning of these neurons, where the firing rate at locations distant from the best direction is lower than at the best direction. We therefore compared the relationship between firing rate and latency across all locations (Figure 8B). We found a strong negative correlation between first-spike latency and firing rate in areas A1 (*r* = −0.67, *p* < 0.01) and CL (*r* = −0.86, *p* < 0.01) of young monkeys. For aged monkeys, regression analysis was hampered because the range of latencies only spanned from 13 to 14 ms, and significant correlations were not seen in either A1 or CL, although these points were consistent with the regression seen in the young animals. These results indicate that the relationship between first-spike latency and firing rate seen in younger animals becomes de-correlated in aged monkeys.

Next, we determined if the population first-spike latencies conveyed structure about spatial tuning. This was quantified by the latency-based spatial tuning index (LTI), which is analogous to the RTI to determine the dynamic range of the latency differences between the best and worst directions. In young monkeys, the population LTI response for neurons in area A1 was 0.11, whereas the population LTI response for neurons in area CL was 0.67. These results suggest that the timing of the population response in area A1 carries little to no temporal information about the location of the stimulus in azimuth, and that the population response in area CL of young monkeys carries broad temporal information about direction in azimuth. In aged monkeys, the population LTI response for neurons in area A1 was 0.13, whereas the population LTI response for neurons in area CL was 0. This result suggests that normal aging degrades the temporal code that is embedded in the timing of population response in area CL.

**Figure 9:**
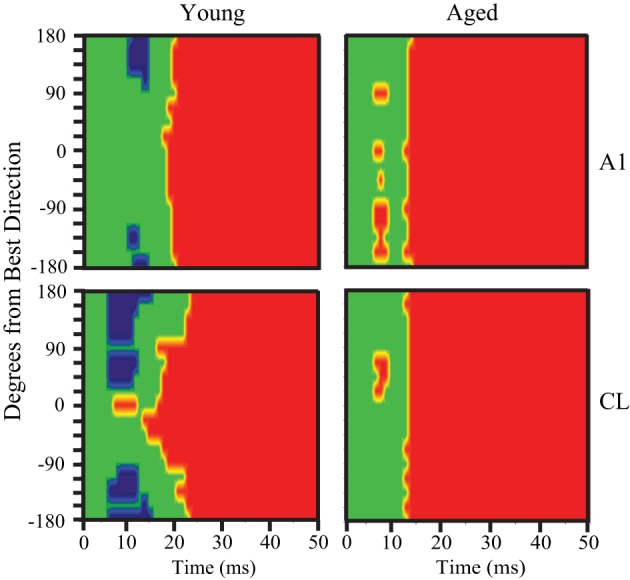
**Pre-onset inhibition in young but not aged monkeys.** Each panel shows the bins that have significantly less (blue) or significantly more (red) activity compared to spontaneous. The population response of young animals is shown at the left, aged animals to the right, with A1 neurons in the top row and CL neurons in the bottom row. A1 of young animals shows pre-onset inhibition at the directions farthest from the best direction, but in CL this is true across a broader spatial extent (bottom left). In contrast, aged animals show no pre-onset inhibition at any direction.

Inspecting the population first-spike tuning curves in young monkeys revealed that the fastest excitatory response was much faster (~8 ms) in CL than what was measured in A1. This was particularly noted in the population responses shown in Figures 2 and 3 when the axis is rotated to illustrate age-differences vertically. This result, along with the temporal tuning results, suggests that the population response in CL may be primed and refined by an initial coordinated volley of inhibitory activity by the tegmental pathway. To test this hypothesis, we re-examined the pre-onset response for significant inhibitory bins in A1 and CL. Figure 9 illustrates significant excitatory and inhibitory bins during the first 50 ms of the population response as a function of the spatial distance from the best direction. Significant excitatory bins are colored in red, significant inhibitory bins are colored in blue, and bins that were not significantly different from spontaneous activity are colored in green. In young monkeys (left column), we found that area CL had significantly more inhibitory bins than area A1 (χ^2^, *p* < 0.05). In A1, we found 14 significant inhibitory bins that flanked the best direction prior to response onset (Figure 9 top left). In CL there was much more inhibitory activity at the flanking locations (Figure 9 bottom left). These results suggest that CL normally receives both a volley of excitation in the best direction and a volley of inhibitory activity that flanks the best direction that arrives simultaneously or slightly preceding the volley of activity into area A1. This implies that a function of the parallel thalamo-cortical pathway into area CL is involved in shaping and refining spatial tuning curves. Interestingly, we did not find a single inhibitory bin prior to the response onset in area A1 or CL of aged monkeys (Figure 9 right column). This indicates that the mechanism of inhibiting the non-best directions is abolished in aged animals, which could account for some, if not all, of the differences in spatial tuning seen in CL between young and aged animals.

## Discussion

**Figure 10:**
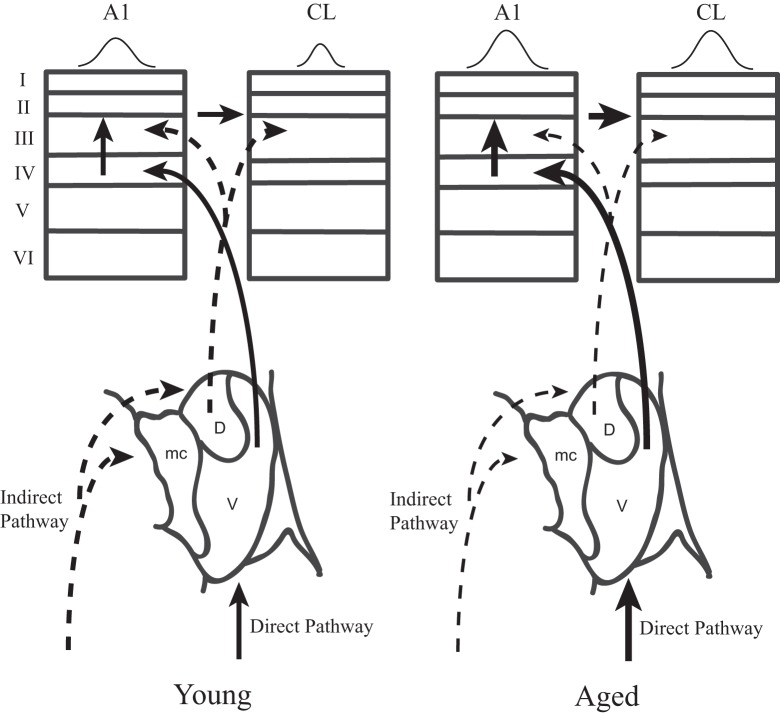
**Two parallel thalamo-cortical circuits to auditory cortex in young (left) and aged (right) monkeys.** Simplified schematic diagram of the ventral and dorsal thalamo-cortical connections to the internal and supragranular layers in the macaque auditory system. The illustration in to lower aspect represents the three main subdivisions of the medial geniculate, the dorsal (D), ventral (V), and medial (mc) divisions. The ventral pathway is from the ventral division to the middle cortical layers of A1 (left) whereas the dorsal pathway initiates in the dorsal division and projects to the upper layers of both A1 and CL. A1 also projects directly to CL. Requiring coincidence of these two pathways is one mechanism by which spatial tuning could be sharpened in CL relative to A1 in young monkeys. In aged monkeys, the increase of activity in A1 and CL degrades with the cortical dynamics that sharpen spatial tuning in CL relative to A1. Abbrevations: V, ventral division of the MGN; D, dorsal division of the MGN; mc, medial division of the MGN; A1, primary auditory area; CL, caudolateral belt area.

This study was motivated by our desire to gain a better understanding of the neurophysiological correlates of the spatial processing deficits that have been reported in aged populations (Brown, 1984; Kubo et al., 1998; Abel et al., 2000), and to extend our recent findings that spatial tuning in area CL becomes degraded by normal aging (Juarez-Salinas et al., 2010). We studied both driven and inhibitory responses during different epochs of the response period to tease apart when in time the spatial tuning was degraded with age and explored whether a lack of inhibition of the parallel thalamic projections to the auditory cortex could account for changes in spatial tuning (Jones, 2003). A schematic of these pathways is shown in Figure 10, where CL neurons receive parallel input from both the dorsal thalamo-cortical pathway as well as serially from A1 [see also Rauschecker et al. (1997)]. Our results indicate that normal aging affects both pathways. They demonstrate that normal aging decreases the efficacy of inhibitory activity to regulate the neuronal response to sound from different locations in space and suggest that the tegmental thalamo-cortical pathway is normally suppressed in young animals. Population rate and latency codes of acoustic space were also impaired by normal aging, which resulted in an age-related change in the population's representation of acoustic space in azimuth (see Figure 3). This lack of inhibition is likely also responsible for the non-monotonic response as a function of time in the aged animals, particularly at nearby but non-best directions, as a function of time (see Figure 2). These results suggest that one of the main central effects of normal aging is the perturbation of the timing and efficacy of inhibition of auditory cortical responses.

## Age-related change in spatial tuning functions

We isolated the effect of normal aging on the basic response properties and spatial tuning functions of neuronal responses in A1 and CL by breaking the neuronal response into different response periods. The finding that there were more neurons inhibited in CL than in A1, and that this inhibitory activity was greater at the flanks of the spatial tuning functions is consistent with inhibitory mechanisms underlying the sharpening of spatial tuning from A1 to CL (Woods et al., 2006). We also noted that while more neurons were inhibited during the late and off periods of the response in both young and aged animals, the overall firing rate differences were greatest during the early period. Previous studies noted that spatial sharpening did not occur, or at least to the same extent, in the mediolateral area (ML) of the belt and were actually worse than neurons in the middle medial area (MM) of the belt in these same young monkeys (Woods et al., 2006). This suggests that inhibitory mechanisms do not play a major role in shaping spatial tuning functions in those belt areas. Previous studies of inhibitory processing in the primate auditory cortex have not addressed this issue, although sideband inhibitory activity is believed to play an important role in shaping the spectral tuning of neurons (e.g., Loftus and Sutter, 2001; O'Connell et al., 2011). Recent evidence suggests that both thalamic and cortical inputs contribute to this inhibitory activity (O'Connell et al., 2011), which is consistent with our finding that the lack of inhibitory activity in aged animals revealed a short-latency input to CL neurons, presumably via the tegmental pathway from the non-lemniscal thalamus. How this inhibition could influence non-spatial processing was not directly tested in these experiments, but presumably age-related differences for such processing would also be seen in these other auditory cortical areas. Evidence from the visual system indicates that motion and orientation processing, also believed to be shaped by inhibitory processes, are also degraded in aged monkeys, suggesting that this is a cortically global phenomenon (Schmolesky et al., 2000; Yu et al., 2006; Yang et al., 2008; Zhang et al., 2008).

The present findings of an age-related increase in driven neuronal activity across the early, late and off periods indicate that auditory cortical neurons in areas A1 and CL are hyperexcitable. While this could be a consequence of increased excitatory drive, it is also consistent with previous studies that found a decline in the efficacy of inhibitory activity to modulate neuronal responses along the auditory pathway [see Caspary et al. (2008)]. Onset firing rates from young monkeys found in this study are consistent with previous studies of auditory cortex in the macaque monkey that reported average discharge rates of ~30 spikes/s in area A1 (Recanzone, 2000). In aged animals, however, onset firing rates were on average three to four times greater, and sustained and off firing rates were on average two to four times greater than the firing rates found in young monkeys. This increased firing rate is consistent with previous studies in rodent auditory brainstem and midbrain (e.g., Finlayson and Caspary, 1993; Palombi and Caspary, 1996; Walton et al., 1997, 2008; Caspary et al., 2005, 2006; Hughes et al., 2010), although it has yet to be determined if complimentary age-related changes of neurons in these auditory nuclei also occur in the primate. Our results are consistent with previous age-related studies that found increased driven activity in the primate visual cortex (Schmolesky et al., 2000; Yu et al., 2006; Yang et al., 2008). These results at the cortical level, coupled with previous studies in other species, strongly suggest that there is concomitant decrease in the efficacy of inhibitory activity in the auditory midbrain and brainstem, presumably as early as the cochlear nucleus, in aged monkeys. Further investigations exploring these early auditory areas in the aged macaque monkeys will help elucidate whether this decline of inhibition throughout the auditory system is a natural aging phenomenon (Caspary et al., 1995; Turner et al., 2005; Walton et al., 2008; Hughes et al., 2010).

## Age-related changes in population response latencies

The increases in driven neuronal activity were complemented by significant age-related changes in the timing of the population response in area CL. In the auditory cortex of young monkeys, the timing of the population excitatory response, as measured by first-spike latencies and the LTI, was spatially tuned. This temporal structure may optimize feed-forward processing of spatial information conveyed to auditory cortex (Van Rullen and Thorpe, 2001). Previously, Woods et al. (2006) used the same data set of young animals and found that only a few neurons in area A1 and CL convey spatial information in the first-spike latency. In the present study, our ability to extract the temporal structure in the timing in the excitatory first-spike can be accounted for by our strategy to normalize space to the best direction and analyze the population response (Miller and Recanzone, 2009) rather than examine the data from non-normalized individual neurons. The population excitatory first-spike temporal code in CL was fairly broad, whereas the population excitatory first-spike temporal code in area A1was non-existent. This finding is consistent with previous studies in the cat that found temporal structure in the coding of space in neurons in area PAF and area DZ (Stecker et al., 2003, 2005).

Four main findings in the present study demonstrate age-related changes in the population response latencies to acoustic spatial stimuli in azimuth. First, the timing of the population excitatory response in area CL of aged monkeys lacked spatial structure. Second, the population excitatory response was significantly faster in A1 and CL of aged monkeys compared to younger animals. Third, the first-spike latency differences between fields were abolished by age. These findings are consistent with Walton et al. (1997) that found that first-spike latencies were faster in low frequency regions of the inferior colliculus in the aged CBA mouse, leading them to suggest that the internal delay mechanism that produced the latency gradient across the tonotopic axis was degraded by age. The results of the present study indicate that the internal delay mechanism that modulates temporal tuning and between field first-spike latency gradients in auditory cortex are also degraded by normal aging. Fourth, population peak latencies in area CL and to a lesser extent in area A1 of aged monkeys were significantly faster than the population peak latencies in A1 and CL of young monkeys. However, the appearance of an oscillatory peak response in both A1 and CL suggests multiple channels in the transmission to auditory cortex. It appears that normal aging abolishes the temporal code that is embedded in the timing of population response in area CL.

In summary, the results of this study indicate that under normal conditions the tegmental input to the caudolateral belt areas is normally inhibited in a balanced manner and must be coupled with input from the core to initiate spiking activity. This inhibition is mainly overcome by inputs representing the best direction, and the flanking inhibition results in sharper spatial tuning of CL neurons compared to A1 neurons. During normal aging, however, this inhibition is lost, giving rise to shorter latencies of CL neurons compared to A1 neurons as the tegmental input can now drive these cells. In addition, the flanking suppression is also lost, giving rise to broader spatial tuning, which presumably could account for the poorer sound localization ability. If this process is generalized throughout auditory cortical pathways, or cortical pathways in general, it predicts that the reversal of this blocked inhibition would restore the normal refinement of stimulus features throughout the hierarchical cortical pathways. Future studies of aged monkeys should focus on experimentally activating or inactivating the tegmental pathway in young and aged monkeys to confirm these findings, and to determine if it is a possible remedial treatment of the symptoms associated with ARHL.

## Author contributions

This manuscript constitutes a novel analysis of previously collected and published data (Woods et al., 2006; Juarez-Salinas et al., 2010). James R. Engle conducted the analysis under the direction of Gregg H. Recanzone. Both authors contributed equally to writing the manuscript.

### Conflict of interest statement

The authors declare that the research was conducted in the absence of any commercial or financial relationships that could be construed as a potential conflict of interest.
